# Rational Design of Flexible, Self-Supporting, and Binder-Free Prussian White/KetjenBlack/MXene Composite Electrode for Sodium-Ion Batteries with Boosted Electrochemical Performance

**DOI:** 10.3390/molecules29133048

**Published:** 2024-06-27

**Authors:** Xiaowen Dai, Jingyun Chun, Xiaolong Wang, Tianao Xv, Zhengran Wang, Chuanliang Wei, Jinkui Feng

**Affiliations:** 1 School of Electrical Engineering, Shandong University, Jinan 250061, China; dxw@mail.sdu.edu.cn (X.D.); wq2103883976@163.com (T.X.); 2 Jiaxing Power Supply Company, State Grid Zhejiang Electric Power Co., Ltd., Jiaxing 314000, China; chunjingyun@163.com; 3 Key Laboratory for Liquid-Solid Structural Evolution & Processing of Materials (Ministry of Education), School of Materials Science and Engineering, Shandong University, Jinan 250061, China; w17812117206@163.com; 4 School of Chemistry and Chemical Engineering, Shandong University, Jinan 250061, China; weichuanliangfly@163.com

**Keywords:** sodium-ion batteries, Ti_3_C_2_T_x_ MXene, Prussian white, energy density

## Abstract

Due to their cost-effectiveness, abundant resources, and suitable working potential, sodium-ion batteries are anticipated to establish themselves as a leading technology in the realm of grid energy storage. However, sodium-ion batteries still encounter challenges, including issues related to low energy density and constrained cycling performance. In this study, a self-supported electrode composed of Prussian white/KetjenBlack/MXene (TK−PW) is proposed. In the TK−PW electrode, the MXene layer is coated with Prussian white nanoparticles and KetjenBlack with high conductivity, which is conducive to rapid Na^+^ dynamics and effectively alleviates the expansion of the electrode. Notably, the electrode preparation method is uncomplicated and economically efficient, enabling large-scale production. Electrochemical testing demonstrates that the TK−PW electrode retains 74.9% of capacity after 200 cycles, with a discharge capacity of 69.7 mAh·g^−1^ at 1000 mA·g^−1^. Furthermore, a full cell is constructed, employing a hard carbon anode and TK−PW cathode to validate the practical application potential of the TK−PW electrode.

## 1. Introduction

With the growing demand for production, energy storage has become a focal point of research in today’s world [1,2]. Rechargeable batteries, known for their flexibility and high energy density, are widely regarded as pivotal components in meeting the development trends in portable electronics and renewable energy systems. In recent decades, lithium-ion batteries have garnered great attention [3,4,5]. However, the increasing cost of lithium-ion batteries, attributed to the scarcity of lithium resources, present a challenge to their widespread adoption in power grid energy storage [6,7,8]. Sodium-ion batteries, sharing similar charge and discharge mechanisms with lithium-ion batteries, benefit significantly from the abundant availability of sodium resources, resulting in a substantial cost reduction. This advantage positions them favorably for extensive applications in the realm of energy storage [9,10]. In addition, large-scale energy storage systems, such as grid storage, do not require as much energy density as portable storage devices [11]. Hence, the utilization of sodium-ion batteries holds great appeal for powering diverse applications, ranging from portable electronic devices to grid-scale energy storage solutions.

Currently, typical sodium-ion battery cathodes consists of transition metal oxides [12,13], polyanionic compounds [14,15], Prussian blue analogs [16,17], and organic compounds [18,19]. PBAs emerge as highly promising cathodes for sodium-ion batteries, characterized by their large lattice gap that can accommodate Na^+^ [20]. Prussian white is a sodium-rich analog of Prussian blue, which can be used to assemble full batteries because of its high sodium content [21]. But Prussian white’s poor cycling performance and electron conductivity hinder the advancements of the energy density of sodium-ion batteries [22]. At present, most research is centered around the synthesis process of Prussian white, which mainly optimizes the material structure by reducing the interstitial water content and lattice defects, thus improving the cyclic stability of the material. For example, Li et al. prepared a Prussian white nanocube with high crystallinity by simple co-precipitation with a capacity retention rate of 78% after 500 cycles [23]. However, the optimized material still needs to be mixed with a binder and coated on a metal collector to prepare the cathode. Non-active materials such as binders and current collectors pose significant obstacles to increasing the energy density of the battery and alleviating volume expansion [24].

MXenes, a kind of transition metal carbides/nitrides/carbonitrides, are currently drawing considerable attention from researchers [25,26]. The chemical formula for MXene is M_n+1_X_n_T_x_ (*n* = 1–4), and M is the transition metal (Ti, V, Nb), X represents either carbon or nitrogen, and T_x_ denotes the surface groups (-F, -OH, -O, and -Cl) [27]. Among them, Ti_3_C_2_T_x_ is the most widely studied MXene variant, known for its ability to form flexible self-supporting electrodes through the assembly of colloidal solutions [28,29]. Although the stacking phenomenon of Ti_3_C_2_T_x_ MXene will reduce its electrochemical performance, doping of nanoparticles has been proved to be an effective approach to prevent the stacking of Ti_3_C_2_T_x_ MXene nanosheets [30]. KetjenBlack(KB) is a cheap conductive agent, which can be doped into the electrode to improve its conductivity effectively [31]. Therefore, the Prussian white/KetjenBlack/MXene composite electrode will be a good choice for increasing battery energy density.

In this paper, we introduce a novel electrode manufacturing process that is simple, time-efficient, and does not necessitate demanding conditions, such as elevated temperatures and high pressure. In the TK−PW electrode, there is no binder, which reduces the pollution of organic matter in the environment. At the same time, Ti_3_C_2_T_x_ MXene and KetjenBlack become conductive links to the Prussian white nanoparticles. The sodium-ion battery, consisting of the TK−PW electrode, demonstrates exceptional cycle stability and rate capabilities. Notably, the TK−PW electrode achieves a discharge capacity of 69.7 mAh·g^−1^ at a current density of 1000 mA·g^−1^. Furthermore, when integrated into a full battery with a hard carbon cathode, the full battery demonstrates favorable cycle stability.

## 2. Results and Discussion

**Figure 1:**
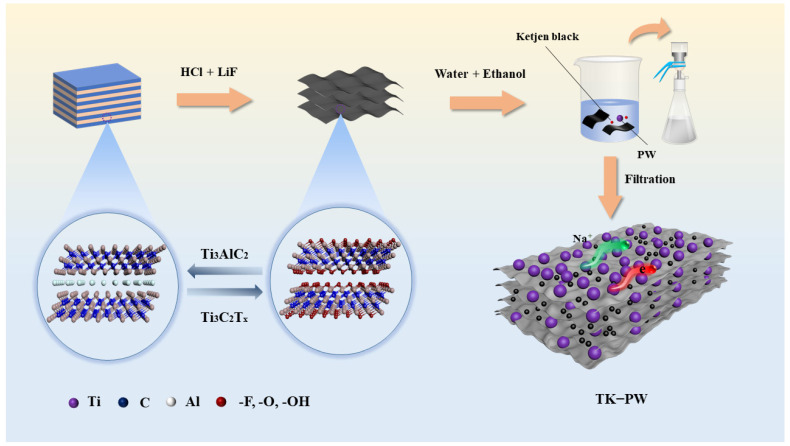
Illustration of the preparation route of TK−PW film.

Figure 1 shows the detailed preparation process of TK−PW film. After Ti_3_AlC_2_ is etched by HCl/LiF, the strong metal bond between Ti-C layers becomes a weak van der Waals force, which makes the Ti_3_C_2_T_x_ MXene layers spacing expand, meaning they can easily be dispersed in aqueous solution [32]. After mixing PW with KetjenBlack and Ti_3_C_2_T_x_ MXene solution for a period of time, PW was dispersed into small nanoparticles mixed between the Ti_3_C_2_T_x_ MXene layers. Then, a large amount of anhydrous ethanol was added to the mixture for redeposition of nanoparticles [33]. The filter paper was covered with a layer of polyethylene film. Due to the hydrophilicity of MXene functional groups, which cannot interact with the hydrophobic PE film, Ti_3_C_2_T_x_ MXene could not adhere to the smooth polyethylene film. Therefore, during the filtration process, some PW nanoparticles were first deposited, and then, the Ti_3_C_2_T_x_ MXene layers and the remaining PW particles were further deposited. TK−PW can be obtained by drying the surface moisture of the filter product in a vacuum oven.

**Figure 2:**
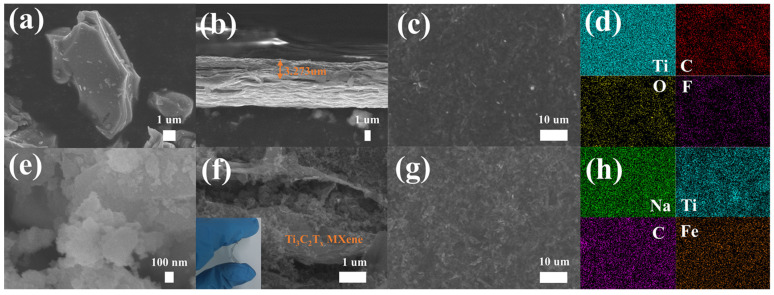
(**a**) SEM image of Ti_3_AlC_2_ MAX phase. (**b**) SEM image of Ti_3_C_2_T_x_ MXene film, side section. (**c**) SEM image of Ti_3_C_2_T_x_ MXene film, front. (**d**) EDS of Ti_3_C_2_T_x_ MXene film. (**e**) SEM image of PW. (**f**) SEM image of TK−PW, side section. (**g**) SEM image of TK−PW, front. (**h**) EDS of TK−PW.

The microtopographies of Ti_3_AlC_2_ and Ti_3_C_2_T_x_ MXenes are shown in Figure 2a,b. Ti_3_AlC_2_ presents an obvious massive structure, while Ti_3_C_2_T_x_ MXenes film presents an obvious lamellar structure with a thickness of about 3.273 um. From the analysis of the EDS test results, the Ti, C, O, and F elements are uniformly distributed on the surface of Ti_3_C_2_T_x_ MXenes (Figure 2c,d). The SEM image and EDS image of PW are shown in Figure 2e and Figure S1a,b. The morphology of PW after grinding by a ball mill presents a large particle composed of many small nanoparticles, with the C, Na, N, and Fe elements uniformly distributed on its surface. In the TK−PW, the Ti_3_C_2_T_x_ MXene layers act as the skeleton of the supporting electrode to fix PW particles and the conductive agent KetjenBlack firmly in the middle of the Ti_3_C_2_T_x_ MXene layers (Figure 2f). At the same time, PW particles expand the distance between the Ti_3_C_2_T_x_ MXene layers and prevent the stacking of Ti_3_C_2_T_x_ MXene layers, thus ensuring the excellent electrical performance of Ti_3_C_2_T_x_ MXene layers [34]. When TK−PW is bent at a certain angle, no cracks appear on its surface, indicating that TK−PW has a certain flexibility. The uniform distribution of the Na, Ti, C, and Fe elements can be observed on the surface of TK−PW, which confirms the successful creation of the composite of Ti_3_C_2_T_x_ MXene and PW (Figure 2g,h). Figure S3a shows the XRD spectra of Ti_3_C_2_T_x_ MXene, PW, and TK−PW. It can be found that the diffraction peaks belonging to both Ti_3_C_2_T_x_ MXene and PW can be found in TK−PW, which proves that TK−PW is a good combination of Ti_3_C_2_T_x_ MXene and PW [35]. In the XRD pattern, the (002) peak shifts significantly to the left, indicating that the strong metal bond between the Ti_3_C_2_T_x_ MXene layers and the aluminum atomic layer is disrupted, resulting in an increase in the interlayer spacing of Ti_3_C_2_T_x_. After PW nanoparticles are added to Ti_3_C_2_T_x_ MXene to form TK−PW, the (002) peak continues to shift to the left, indicating that the nanoparticles prevent Ti_3_C_2_T_x_ MXene stacking. From the full spectrum of the XPS, it can be observed that there is a uniform distribution of Na (belonging to PW), Ti (belonging to Ti_3_C_2_T_x_ MXene), and C elements on the surface of TK−PW (Figure S3b).

**Figure 3:**
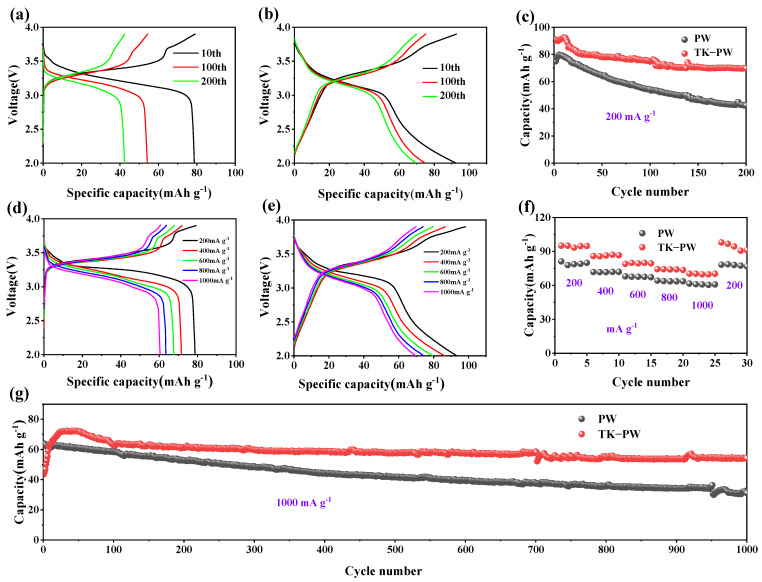
Charge/discharge profiles of (**a**) PW and (**b**) TK−PW at the 10th, 100th, and 200th cycles. (**c**) Cycling stability at 200 mA·g^−1^ for PW and TK−PW. Charge/discharge profiles of (**d**) PW and (**e**) TK−PW at current densities ranging from 200 mA·g^−1^ to 1000 mA·g^−1^. (**f**) Rate performance at various current densities. (**g**) Cycling stability at 1000 mA·g^−1^ for PW and TK−PW.

The discharge/charge voltage profiles of the PW and TK−PW cathodes at a rate of 200 mA·g^−1^ are shown in Figure 3a,b. The discharge capacities of the PW electrode at the 10th, 100th, and 200th cycles were found to be 78.7, 54.2, and 42.4 mAh·g^−1^ (Figure 3a), respectively. Similarly, the discharge capacities of the TK-PW electrode at the same cycles were 92.5, 74.8, and 69.7 mAh·g^−1^, respectively (Figure 3b). We can see that the electrochemical platform of the TK−PW electrode exhibited minor change with increasing cycles, indicating a lower polarization degree compared to the PW electrode. The charge and discharge curves of the TK−PW electrode appeared smoother than the PW electrode, which can be attributed to most of the PW particles being wrapped in the MXene [36]. After 200 cycles, the PW electrode’s discharge capacity decreases to 42.3 mAh·g^−1^, retaining 56.5% of its initial capacity (Figure 3c). The TK−PW electrode’s capacity decreases to 69.7 mAh·g^−1^, with a high retention rate of 74.9%, indicating that the TK−PW electrode exhibits better cyclic stability. At current densities of 200, 400, 600, 800, and 1000 mA·g^−1^, the discharge capacities of the PW electrode are 78.7, 71.6, 67.6, 63.5, and 60.7 mAh·g^−1^, respectively (Figure 3d). The specific capacities of the TK-PW electrode at the same current densities are 93.0, 86.3, 79.4, 74.1, and 69.7 mAh·g^−1^, respectively (Figure 3e). At a current density of 1000 mA·g^−1^, the PW electrode exhibits a discharge capacity that is 77.1% of the initial capacity, whereas the TK−PW electrode shows a capacity of 74.9% relative to its initial capacity. The TK−PW electrode exhibits a slightly lower capacity retention rate compared to the PW electrode, possibly due to the lack of PVDF in the TK−PW electrode, which results in less contact between the conductive agent and the active substance. Nevertheless, the specific capacity of the TK−PW electrode remains higher than that of the PW electrode at high currents. Furthermore, when the current density returns to 200 mA·g^−1^, the discharge capacity of the TK−PW electrode is almost the same as the initial capacity, demonstrating the good reversibility of the electrode (Figure 3f). Additionally, the long-term performance of the PW electrode and TK−PW electrode is compared at a high current density of 1000 mA·g^−1^. After 1000 cycles of charge and discharge, the PW electrode’s specific capacity is 31.3 mAh·g^−1^, while that of the TK−PW electrode is 54.1 mAh·g^−1^. Moreover, the cycle stability of the TK−PW electrode is significantly better than that of the PW electrode. It is worth noting that during the initial stage of the electrochemical cycle of the TK−PW electrode, there is a notable increase in capacity, which can be ascribed to the activation process resulting from the infiltration of the electrolyte into the electrode [37].

**Figure 4:**
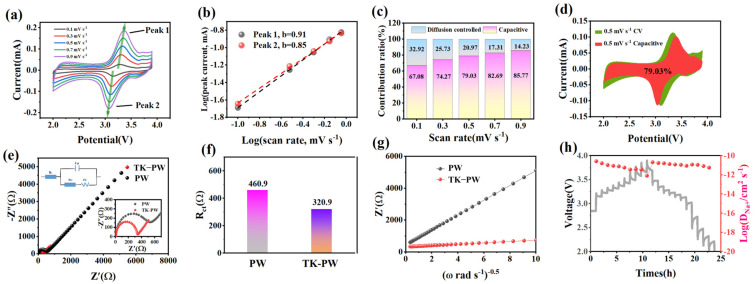
Electrochemical kinetics and sodium storage performance of TK−PW electrode. (**a**) CV curves ofTK−PW at various scan rates from 0.1 to 0.9 mV s^−1^. (**b**) Analysis of b values of Peak 1 and Peak2. (**c**) Diffusion and capacitive contribution ratios at various scan rates. (**d**) Pseudocapacitive fitting at 0.5 mV·s^−1^ for TK−PW (The red area represents theoretical pseudocapacitive capacity; the green area represents actual capacitive capacity on the CV curve). (**e**) Nyquist plots of PW and TK−PW (inset: the equivalent circuit modeling). (**f**) Charge transfer resistance of PW and TK−PW. (**g**) Linear relationship between ω^−1/2^ and Z_w_ in the Warburg region. (**h**) GITT curves (Left axis: Grey curve) and Na^+^ diffusion coefficients (Right axis: Red curve).

CV, EIS, and GITT tests were conducted to determine the kinetics of Na^+^ storage after cycling. Figure 4a shows the CV curves of the TK−PW electrode at scan rates ranging from 0.1 mV·s^−1^ to 0.9 mV·s^−1^. As the scan rate increases, due to electrode polarization, the oxidation peak moves towards higher voltage, and the reduction peak shifts towards a lower voltage. The peak current on the CV curve can be described by Equation (1):

(1) $$i=a\cdot v^{b}$$

where a and b are constants and v is the scan rate. The b value for peak 1 is 0.91, and the b value for peak 2 is 0.85, both falling within the range of 0.5 to 1, especially, the b value is close to 1. This suggests the occurrence of redox reactions and capacitive processes during the charge–discharge cycle, as illustrated in Figure 4b [38]. The contribution ratios of the capacitance at different scan rates is as follows: 67.08%, 74.27%, 79.03%, 82.69%, and 85.77% at scan rates of 0.1, 0.3, 0.5, 0.7, and 0.9 mV·s^−1^, respectively (Figure 4c). Figure 4d illustrates the calculated capacitance ratio of TK−PW at a scan rate of 0.5 mV·s^−1^. The cyclic voltammetry reveals that the superior performance of the TK−PW electrode is primarily attributable to the pseudocapacitance effect resulting from the nanometer-sized PW particles in the electrode [39]. EIS analysis was performed on the two electrodes after cycling. The fitted Nyquist plot of the PW electrode and TK−PW electrode after cycling is shown in Figure 4e. In the equivalent circuit in the upper left corner, Rs represents electrolyte resistance, Cdl represents a double-layer capacitor, Rct represents charge transfer resistance (area of semicircle region), and Zw is diffusion-related impedance (slope of incline line) [29]. The Rct of the PW electrode is 460.9 Ω, while the Rct of the TK−PW electrode is 320.9 Ω, according to the Nyquist plots of the high-frequency area (Figure 4e,f). Figure 4g illustrates the linear relationship between impedance at low frequencies and the inverse square root of the angular frequency on the Nyquist plot, serving to characterize the diffusion properties of ions [40]. The smaller the slope, the larger value of $D_{{Na}^{+}}$ indicating better Na^+^ transfer kinetics, confirming the improved high current discharge capacity of the TK−PW electrode [41]. The GITT technique was used to further evaluate the diffusion process of Na^+^ in the TK−PW electrode in Figure 4h. The ${Na}^{+}$ diffusion coefficient can be determined by the equation

(2) $$D_{Na^{+}}=\frac{1}{2}{\left[\left(\frac{V_{m}}{SF\sigma }\right)\frac{\delta E}{\delta x}\right]}^{2}$$

where σ is the slope of the Warburg impedance Z_w_ and ω^−1/2^, S is the contact area between the electrolyte and electrode, and F represents the Faraday constant [42]. The $D_{Na^{+}}$ for the TK−PW electrode ranges between 10^−12^ and 10^−10^ cm^2^·s^−1^, better than that of the PW electrode. Therefore, it can be inferred that the MXene structure provides a larger contact area and a fast pathway for ion transfer for nanoparticles.

**Figure 5:**
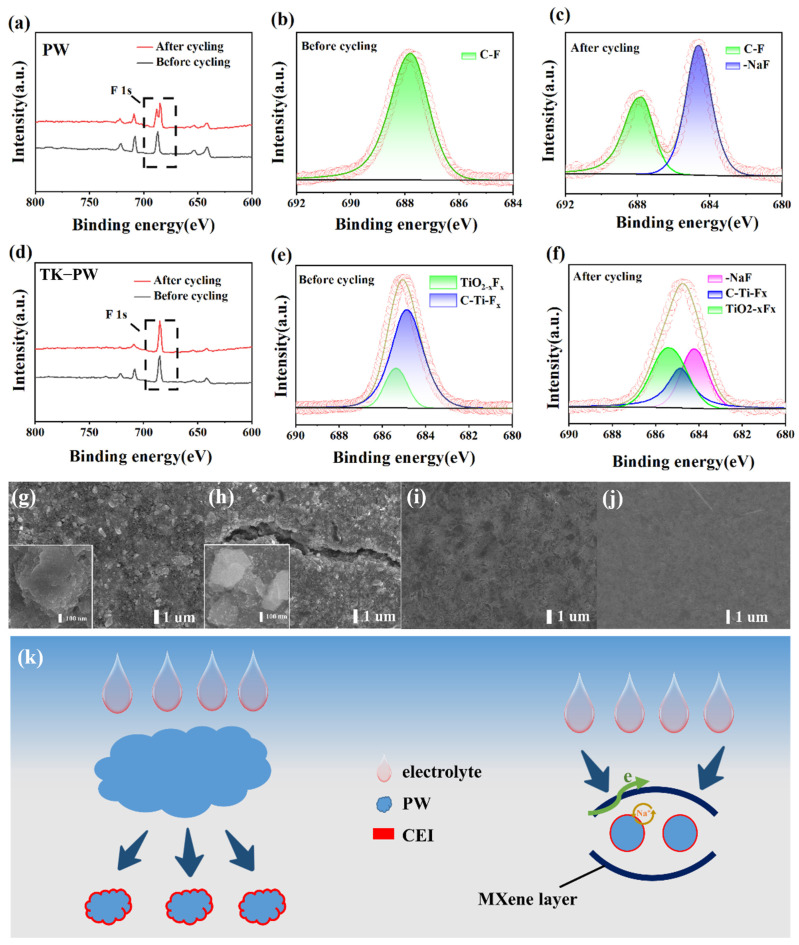
(**a**) Full spectrum of XPS for PW cathodes before and after cycling. F 1s XPS spectra of PW (**b**) before cycling and (**c**) after cycling (red circles represents cumulative peak fitting). (**d**) Full spectrum of XPS for TK−PW cathodes before and after cycling. F 1s XPS spectra of TK−PW (**e**) before cycling and (**f**) after cycling. SEM image of PW (**g**) before cycling and (**h**) after cycling (inset: high resolution SEM images). SEM image of TK−PW (**i**) before cycling and (**j**) after cycling. (**k**) Schematic diagram of the electrochemical mechanism during the PW and TK−PW cathodes’ cycling process.

The chemical composition of the cathode/electrode interfaces (CEIs) of the PW electrode and TK−PW electrode were analyzed by XPS. As can be seen from the full spectrum of XPS of the electrodes before and after the cycle, a set of new peaks was added to F 1s of the PW electrode, while the peak height and peak width of F 1s of the TK−PW electrode changed (Figure 5a,d). Prior to the electrochemical process, there is a fitted peak in the spectrum of the PW electrode, corresponding to 687.9 eV of PVDF (Figure 5b) [43]. After the electrochemical reaction, the F 1s spectrum is divided into two peaks. In addition to the original C-F peak, a new NaF peak at 684.2 eV is added (Figure 5c) [44]. For the TK−PW electrode, before the electrochemical reaction, the F 1s spectrum can be divided into two peaks, one is the C-Ti-F_x_ peak at 684.9 eV, the other is the TiO_2-x_F_x_ peak at 685.4 eV, both of which are derived from the Ti_3_C_2_T_x_ MXene (Figure 5e) [45]. After the electrochemical reaction of the TK−PW electrode, an NaF peak also appeared. NaF-containing CEIs reduce the electrolyte’s tendency to erode the active ingredient [42]. In our other work, the control electrode did not detect the appearance of an NaF peak after the electrochemical reaction, which may be attributed to the limited amount of NaF due to the short cycle charge and discharge times of the battery and the relatively thin CEI itself [44]. A small amount of NaF was detected in the experimental electrode, which was due to the high specific surface area of the nanoparticles and the full contact with the electrolyte. The change in the composition of the substance was analyzed by the peak area of the XPS peak [45]. At the early stage of the electrochemical cycle, the NaF content of the PW electrode changes very little, while at the late stage of the electrochemical cycling, the NaF content increases sharply, which indicates that as the electrochemical reaction progresses, the volume expansion of the Prussian white particles causes them to break into smaller particles, exposing the fresh PW surface to the organic electrolyte and forming a new CEI film [41,46]. For the TK−PW electrode, the content of NaF increases gently during the electrochemical reaction (Figure 5f). Ti_3_C_2_T_x_ MXene, distinguished by its high flexibility, partnered with nanoparticles having an elevated specific surface area, can slow the volume expansion of active substances, making it suitable for the rapid insertion and removal of Na^+^ [47,48]. In addition, the CEI film covered with PW nanoparticles prevents the organic electrolyte from eroding the active substance and ensures its good cycling stability [42]. The microscopic appearance of the electrode before and after the cycle can be observed by SEM. A layer of thick and elongated cracks appears on the PW electrode surface after cycling, indicating pronounced expansion of the PW electrode (Figure 5g,h). The TK−PW electrode maintains a smooth surface after circulation, with no visible cracks, indicating minimal expansion of the electrode (Figure 5i,j). Figure 5k shows the cycling mechanism of the two types of electrodes. During cycling, the PW electrode undergoes significant volume expansion, resulting in fragmentation of active particles into smaller ones and erosion by organic solvents. The TK−PW electrode, incorporating the highly flexible Ti_3_C_2_T_x_ MXene, effectively mitigates the volume swelling of the active substance during cycling. The CEI on the surface of the PW nanoparticles prevents electrolyte erosion and enhances the cycle stability of the active substance. Additionally, the conductive framework formed by Ti_3_C_2_T_x_ MXene and KetjenBlack facilitates efficient electron transfer. The close contact between the PW nanoparticles and electrolyte creates abundant Na^+^ migration paths, promoting rapid Na^+^ dynamics.

**Figure 6:**
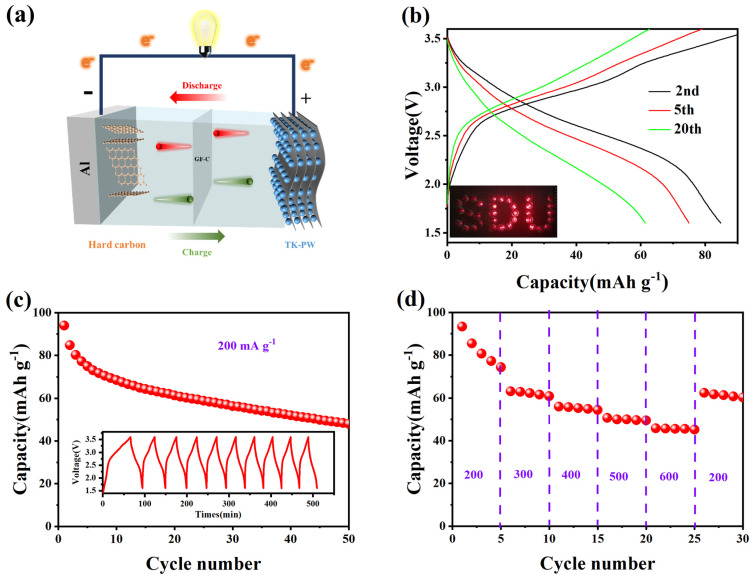
Electrochemical performance of full cells based on TK−PW cathode and hard carbon anode. (**a**) The full battery model. (**b**) Charge/discharge curve at 200 mA·g-1 current density (insert: a battery-powered cluster of LEDs arranged in the shape of an “SDU”). (**c**) Cycling stability at 200 mA·g^−1^ (insert: the charge/discharge platform of the full cell). (**d**) Rate performance of the full battery from 200 mA·g^−1^ to 600 mA·g^−1^.

The Prussian white, which is abundant in sodium, is expected to react with the hard carbon anode to form a high-performance full battery. Figure 6a illustrates the structure of the full battery, and the experimental section provides detailed information on its preparation. The electrochemical performance of the full battery was evaluated across a voltage range of 1.6 V to 3.6 V. Prior to the charge and discharge tests of the full battery, the TK−PW cathode and hard carbon anode were pre-modified in the half battery for 3 cycles. Notably, a home-patterned “SDU” array consisting of LEDs was successfully illuminated, demonstrating its potential for various applications (inset of Figure 6b). After 50 cycles, the capacity retention rate of the full battery reached 51.6% (Figure 6c), and a charge–discharge cycle could be accomplished in approximately 46 min (inset of Figure 6c). Furthermore, the full cell exhibited excellent rate capacity, with reversible capacities of 80, 62.2, 55.3, 50.0, and 45.3 mAh·g^−1^ at current densities of 200, 300, 400, 500, and 600 mA·g^−1^, respectively (Figure 6d).

## 3. Conclusions

In summary, we developed a simple filtration method to prepare self-supporting electrodes without adhesives and other non-active components. This approach can alleviate the weight of the battery to some extent and improve its energy density. Given the larger interlayer spacing of Ti_3_C_2_T_x_ MXene, it mitigates the volume expansion caused by ion insertion and extraction during the charge and discharge process. Additionally, Prussian white nanoparticles effectively prevent MXene stacking and provide a fast Na^+^-transfer channel. Thus, the prepared TK−PW electrode exhibits excellent rate capability and a long cycle life. Table 1 summarizes and compares the electrochemical performance of the TK−PW and PW electrodes as well as similar electrode studies on sodium-ion batteries. Specifically, the TK−PW electrode retains 74.9% of its capacity after 200 cycles at current density of 200 mA·g^−1^, and its discharge capacity at a current density of 1000 mA·g^−1^ is 69.7 mAh·g^−1^. The full cell, consisting of a hard carbon anode and TK−PW cathode, retains 51.6% of its capacity after 50 cycles at a current density of 200 mA·g^−1^, demonstrating the practical application potential of the TK−PW.

## 4. Experimental Methods

### 4.1. Preparation of Ti_3_C_2_T_x_ MXene Film

Ti_3_C_2_T_x_ MXene film is prepared using a mild HCl/LiF etching method [55]. A volume of 20 mL of 1.2 mol/L concentrated hydrochloric acid was diluted with 4 mL of H_2_O, and then, reacted with 1.6 g of LiF in a Teflon reactor for 5 min. Then, the 1 g of Ti_3_AlC_2_ MAX phase was added and the reactor was stirred in a water bath at 36.5 °C for 36 h. The etched products were taken out, put into a centrifugal tube, washed until obviously expanded, and then, water was added and shaken to obtain the Ti_3_C_2_T_x_ MXene colloidal solution. The Ti_3_C_2_T_x_ MXene suspension concentrationc was about 2.2 mg/mL. The 20 mL of Ti_3_C_2_T_x_ MXene colloidal solution was filtered, and after the water on the surface of the filtered product evaporated, the Ti_3_C_2_T_x_ MXene film was formed. The preparation of thin films is based on our previous research findings [33].

### 4.2. Preparation of TK−PW Film

Prussian white (Na_1.4_Fe[Fe(CN)_6_]) was purchased from Sinopharm Chemical Reagent Co., Ltd., Shanghai, China. Put 16 mg of Prussian white (PW) into a ball mill and grind for 1 h. Next, mix it with 16 mg of superconducting carbon black (KetjenBlack), 4 mL of Ti_3_C_2_T_x_ MXene colloidal solution, and 16 mL of water. Stir the mixture for 30 min. Then, add 180 mL of anhydrous ethanol and stir for 5 min. Filter the mixture using a polyethylene diaphragm on the filter paper. Put the filter product into a 50° vacuum oven for 5 h to obtain TK−PW film.

### 4.3. Materials Characterization

The physical components of the composite were analyzed using XRD diffraction (XRD, MiniFlex 600, Cu-Kα radiation, Tokyo, Japan). The material’s chemical composition was examined through X-ray photoelectron spectroscopy (XPS, Axis Supra, Al-Kα source, Japan). The microscopic morphology of the substance was observed using a scanning electron microscope (SEM, JSM-7610F, Tokyo, Japan). The element distribution on the material surface was acquired using energy dispersive spectrometer (EDS, X-max, Oxford, UK).

### 4.4. Electrochemical Characterization

The TK−PW was cut into discs with a diameter of 10 mm, which served as the cathode of the sodium-ion battery. The electrolyte used was NaClO_4_ with ethylene carbonate (EC) and diethyl carbonate (DEC) (1:1 by volume), followed by the addition of 10% fluoroethylene carbonate (FEC). Each TK−PW electrode had an active material load of approximately 0.5 mg. In detail, the TK−PW electrode was assembled with a glass-fiber diaphragm (16 mm diameter), and sodium foil (14.4 mm diameter) inside an Ar-filled glove box. For the basic PW-Na batteries, the PW electrode contained 70 wt% active material, 20 wt% Super-p, and 10 wt% polyvinylidene fluoride (PVDF), which were mixed in N-methyl-2-pyrrolidone (NMP) for 24 h. The mixture was evenly coated on aluminum foil, and then, placed in a vacuum oven at 80 °C for 8 h. All electrodes were cut to small plates with a diameter of 10 mm, and each had an active material load of approximately 0.7–0.9 mg/cm^2^. Coin-type (CR2032) cells were assembled in an Ar-filled glove box, with the same electrolyte, diaphragm, and Na anode as the TK−PW-Na batteries. The charge/discharge performance of the battery was assessed using a battery system (CT-4008 Netware) within the potential range of 2.0 to 3.9 V. Cyclic voltammetry (CV) curves (between 2.0 and 3.9 V) and electrochemical impedance spectra (EIS) (frequency of 0.01–106 Hz) were evaluated by a CHI660E electrochemical workstation with various scan rates.

## Figures and Tables

**Table 1:** Summary of electrochemical performance for TK−PW and PW electrodes as well as similar electrode studies on sodium-ion batteries.

Electrode Material	Preparation Method	Rate Capability		Cycle Performance		Ref.
		Current Density (mA·g^−1^)	Capacity (mAh·g^−1^)	Cycle Number	Capacity Retention (mAh·g^−1^)	
TK−PW	Vacuum Filtration Method	200	93	1000	54.1 at 1000 mA·g^−1^	This work
PW	Coating Method	200	78.7	1000	31.3 at 1000 mA·g^−1^	This work
PW-1	Coating Method	300 (2 C)	108	700	87.1 at 0.5 C	[49]
PW-2	Coating Method	300 (2 C)	110	580	65.3 at 0.5 C	[49]
PW-HN	Coating Method	240 (2 C)	115	5000	80% at 10 C	[50]
Na_1.58_Fe[Fe(CN)_6_]_0.92_	Coating Method	340 (2 C)	113	800	90% at 2 C	[51]
NMHFCs	Coating Method	102 (0.85 C)	92	30	121 at 1/20 C	[52]
PB-1	Coating Method	20	89.5	400	81.45 at 20 mA·g^−1^	[53]
PB@C	Coating Method	2000 (20 C)	100	1000	100 at 2000 mA·g^−1^	[54]

## Data Availability

Data are contained within the article and Supplementary Materials.

## Supplementary Materials

The Supporting Information is available free of charge at https://www.mdpi.com/article/10.3390/molecules29133048/s1. Figure S1: SEM and EDS of PW. Figure S2: The XRD of Ti_3_C_2_T_x_ and Ti_3_AlC_2_. Figure S3: The XRD and XPS of PW and TK−PW.

## References

[1] Kandari R., Neeraj N., Micallef A. (2022). Review on recent strategies for integrating energy storage systems in microgrids. Energies.

[2] Hjalmarsson J., Thomas K., Boström C. (2023). Service stacking using energy storage systems for grid applications–A review. J. Energy Storage.

[3] Huang B., Pan Z., Su X., An L. (2018). Recycling of lithium-ion batteries: Recent advances and perspectives. J. Power Sources.

[4] Ma S., Jiang M., Tao P., Song C., Wu J., Wang J., Deng T., Shang W. (2018). Temperature effect and thermal impact in lithium-ion batteries: A review. Prog. Nat. Sci. Mater. Int..

[5] Yao Y., Zhu M., Zhao Z., Tong B., Fan Y., Hua Z. (2018). Hydrometallurgical Processes for Recycling Spent Lithium-Ion Batteries: A Critical Review. ACS Sustain. Chem. Eng..

[6] Xu M., Liu M., Yang Z., Wu C., Qian J. (2022). Research Progress on Presodiation Strategies for High Energy Sodium-Ion Batteries. Acta Phys. Chim. Sin..

[7] Huang Z.-X., Gu Z.-Y., Heng Y.-L., Huixiang Ang E., Geng H.-B., Wu X.-L. (2023). Advanced layered oxide cathodes for sodium/potassium-ion batteries: Development, challenges and prospects. Chem. Eng. J..

[8] Lin X.-M., Yang X.-T., Chen H.-N., Deng Y.-L., Chen W.-H., Dong J.-C., Wei Y.-M., Li J.-F. (2023). In situ characterizations of advanced electrode materials for sodium-ion batteries toward high electrochemical performances. J. Energy Chem..

[9] Zhang L., Li X., Yang M., Chen W. (2021). High-safety separators for lithium-ion batteries and sodium-ion batteries: Advances and perspective. Energy Storage Mater..

[10] Lee H.W., Wang R.Y., Pasta M., Woo Lee S., Liu N., Cui Y. (2014). Manganese hexacyanomanganate open framework as a high-capacity positive electrode material for sodium-ion batteries. Nat. Commun..

[11] Oz E., Altin S., Avci S. (2023). Investigation of physical and electrochemical properties of Ni-doped Tunnel/P2 hybrid Na_0.44_MnO_2_ cathode material for sodium-ion batteries. J. Solid State Chem..

[12] Liu Q., Hu Z., Li W., Zou C., Jin H., Wang S., Chou S., Dou S.-X. (2021). Sodium transition metal oxides: The preferred cathode choice for future sodium-ion batteries?. Energy Environ. Sci..

[13] Pahari D., Kumar A., Das D., Puravankara S. (2022). P2-type Na_x_TmO_2_ oxides as cathodes for non-aqueous sodium-ion batteries—Structural evolution and commercial prospects. Int. J. Energy Res..

[14] Lv Z., Ling M., Yue M., Li X., Song M., Zheng Q., Zhang H. (2021). Vanadium-based polyanionic compounds as cathode materials for sodium-ion batteries: Toward high-energy and high-power applications. J. Energy Chem..

[15] Wu H., Chen Y., Wen T., Chen L., Pu X., Chen Z. (2023). Advances in Vanadium-Redoxed Polyanions for High-Voltage Sodium-Ion Batteries. Batteries.

[16] Lu Y., Wang L., Cheng J., Goodenough J.B. (2012). Prussian blue: A new framework of electrode materials for sodium batteries. Chem. Commun..

[17] Xie B., Sun B., Gao T., Ma Y., Yin G., Zuo P. (2022). Recent progress of Prussian blue analogues as cathode materials for nonaqueous sodium-ion batteries. Coord. Chem. Rev..

[18] Wang H.g., Zhang X.b. (2018). Organic carbonyl compounds for sodium-ion batteries: Recent progress and future perspectives. Chem.–A Eur. J..

[19] Zhao Q., Whittaker A.K., Zhao X.S. (2018). Polymer Electrode Materials for Sodium-ion Batteries. Materials.

[20] Fu H., Xia M., Qi R., Liang X., Zhao M., Zhang Z., Lu X., Cao G. (2018). Improved rate performance of Prussian blue cathode materials for sodium ion batteries induced by ion-conductive solid-electrolyte interphase layer. J. Power Sources.

[21] Lim C.Q.X., Tan Z.-K. (2021). Prussian White with Near-Maximum Specific Capacity in Sodium-Ion Batteries. ACS Appl. Energy Mater..

[22] Nielsen I., Dzodan D., Ojwang D.O., Henry P.F., Ulander A., Ek G., Häggström L., Ericsson T., Boström H.L.B., Brant W.R. (2022). Water driven phase transitions in Prussian white cathode materials. J. Phys. Energy.

[23] Li C., Zang R., Li P., Man Z., Wang S., Li X., Wu Y., Liu S., Wang G. (2018). High Crystalline Prussian White Nanocubes as a Promising Cathode for Sodium-ion Batteries. Chem. Asian J..

[24] Song W.-L., Li X., Fan L.-Z. (2016). Biomass derivative/graphene aerogels for binder-free supercapacitors. Energy Storage Mater..

[25] Ma P., Fang D., Liu Y., Shang Y., Shi Y., Yang H.Y. (2021). MXene-Based Materials for Electrochemical Sodium-Ion Storage. Adv. Sci..

[26] Lei Y.-J., Yan Z.-C., Lai W.-H., Chou S.-L., Wang Y.-X., Liu H.-K., Dou S.-X. (2020). Tailoring MXene-Based Materials for Sodium-Ion Storage: Synthesis, Mechanisms, and Applications. Electrochem. Energy Rev..

[27] Xu H., Fan J., Pang D., Zheng Y., Chen G., Du F., Gogotsi Y., Dall’Agnese Y., Gao Y. (2022). Synergy of ferric vanadate and MXene for high performance Li- and Na-ion batteries. Chem. Eng. J..

[28] Yang J., Wang T., Guo X., Sheng X., Li J., Wang C., Wang G. (2021). Flexible sodium-ion capacitors boosted by high electrochemically-reactive and structurally-stable Sb_2_S_3_ nanowire/Ti_3_C_2_T_x_ MXene film anodes. Nano Res..

[29] Sun N., Zhu Q., Anasori B., Zhang P., Liu H., Gogotsi Y., Xu B. (2019). MXene-Bonded Flexible Hard Carbon Film as Anode for Stable Na/K-Ion Storage. Adv. Funct. Mater..

[30] Xiao S., Zhang X., Zhang J., Wu S., Wang J., Chen J.S., Li T. (2018). Enhancing the lithium storage capabilities of TiO_2_ nanoparticles using delaminated MXene supports. Ceram. Int..

[31] Yang C., Wu Q., Cao Y., Gao Y., Li A., Liu X., Zhang X., Tian Z., Liu R. (2021). α-MnO_2_/super-P with conductive carbon network for rechargeable aqueous Zinc ion batteries. Mater. Lett..

[32] Murali G., Reddy Modigunta J.K., Park Y.H., Lee J.H., Rawal J., Lee S.Y., In I., Park S.J. (2022). A Review on MXene Synthesis, Stability, and Photocatalytic Applications. ACS Nano.

[33] Chun J., Wang X., Wei C., Wang Z., Zhang Y., Feng J. (2023). Flexible and free-supporting Prussian blue analogs/MXene film for high-performance sodium-ion batteries. J. Power Sources.

[34] Zhang W.X., Zhang J.H., Zhang Y.K., He C., Zhao P. (2022). NiS_2_ nanoparticles anchored on MXene conductive frameworks with enhanced lithium and sodium storage properties. Ionics.

[35] Wei C., Xi B., Wang P., Wang Z., An X., Li Y., Feng J., Xiong S. (2024). Rapid Growth of Bi_2_Se_3_ Nanodots on MXene Nanosheets at Room Temperature for Promoting Sulfur Redox Kinetics. Inorg. Chem..

[36] Wang Z., Zhang Y., Jiang H., Wei C., An Y., Tan L., Xiong S., Feng J. (2023). Free-standing Na_2_C_6_O_6_/MXene composite paper for high-performance organic sodium-ion batteries. Nano Res..

[37] Wang W., Zhang P., Li S., Zhou C., Guo S., Liu J., Zhou J., Jian X., Yang Y., Lei Y. (2020). Nitrogen-doped carbon-wrapped porous FeMnO_3_ nanocages derived from etched prussian blue analogues as high-performance anode for lithium ion batteries. J. Power Sources.

[38] Chen Y., Woo H.J., Syed Mohd Fadzil S.A.F., Tan W., Wang F., Mohd Arof A.K. (2022). Cage-Like Porous Prussian Blue as High-Capacity Cathode for Sodium-Ion Batteries. ACS Appl. Nano Mater..

[39] Cheng F., Liang J., Tao Z., Chen J. (2011). Functional materials for rechargeable batteries. Adv. Mater..

[40] Li L., Zheng H., Wang S., Chen X., Yang S., Feng C. (2022). Construction of Na_3_V_2_(PO_4_)_3_/C nanoplate as cathode for stable sodium ion storage. Ionics.

[41] Xie B., Wang L., Li H., Huo H., Cui C., Sun B., Ma Y., Wang J., Yin G., Zuo P. (2021). An interface-reinforced rhombohedral Prussian blue analogue in semi-solid state electrolyte for sodium-ion battery. Energy Storage Mater..

[42] Ye M., You S., Xiong J., Yang Y., Zhang Y., Li C.C. (2022). In-situ construction of a NaF-rich cathode–electrolyte interface on Prussian blue toward a 3000-cycle-life sodium-ion battery. Mater. Today Energy.

[43] Chun J., Wang X., Zhang Y., Wei C., Wang Z., Feng J. (2023). Ti_3_C_2_T_x_ film current collectors for high-performance sodium-ion batteries. Vacuum.

[44] Zhang N., Wang B., Jin F., Chen Y., Jiang Y., Bao C., Tian J., Wang J., Xu R., Li Y. (2022). Modified cathode-electrolyte interphase toward high-performance batteries. Cell Rep. Phys. Sci..

[45] You Y., He Z. (2023). Phenol degradation in iron-based advanced oxidation processes through ferric reduction assisted by molybdenum disulfide. Chemosphere.

[46] ur Rehman W., Jiang Z., Qu Z., Ahmed S., Ghani A., Xu Y. (2022). Highly crystalline Prussian blue cubes filled with tin oxide as anode materials for lithium-ion batteries. Appl. Surf. Sci..

[47] Fu B., Man H., Zhao J., Wang F., Fang F., Sun D., Song Y. (2022). Less Is More: High-Performance All-Solid-State Electrode Enabled by Multifunctional MXene. ACS Appl. Energy Mater..

[48] Ye W., Yu L., Sun M., Cheng G., Fu S., Peng S., Han S., Yang X. (2019). Yolk−shell Prussian blue analogues hierarchical microboxes: Controllably exposing active sites toward enhanced cathode performance for lithium ion batteries. Electrochim. Acta.

[49] Shen Z., Guo S., Liu C., Sun Y., Chen Z., Tu J., Liu S., Cheng J., Xie J., Cao G. (2018). Na-rich Prussian white cathodes for long-life sodium-ion batteries. ACS Sustain. Chem. Eng..

[50] Ren W., Zhu Z., Qin M., Chen S., Yao X., Li Q., Xu X., Wei Q., Mai L., Zhao C. (2019). Prussian white hierarchical nanotubes with surface-controlled charge storage for sodium-ion batteries. Adv. Funct. Mater..

[51] Tang X., Liu H., Su D., Notten P.H., Wang G. (2018). Hierarchical sodium-rich Prussian blue hollow nanospheres as high-performance cathode for sodium-ion batteries. Nano Res..

[52] Wang L., Lu Y., Liu J., Xu M., Cheng J., Zhang D., Goodenough J.B. (2013). A superior low-cost cathode for a Na-ion battery. Angew. Chem..

[53] Li W.-J., Chou S.-L., Wang J.-Z., Kang Y.-M., Wang J.-L., Liu Y., Gu Q.-F., Liu H.-K., Dou S.-X. (2015). Facile method to synthesize Na-enriched Na_1+x_FeFe(CN)_6_ frameworks as cathode with superior electrochemical performance for sodium-ion batteries. Chem. Mater..

[54] Jiang Y., Yu S., Wang B., Li Y., Sun W., Lu Y., Yan M., Song B., Dou S. (2016). Prussian blue@ C composite as an ultrahigh-rate and long-life sodium-ion battery cathode. Adv. Funct. Mater..

[55] Zhang T., Pan L., Tang H., Du F., Guo Y., Qiu T., Yang J. (2017). Synthesis of two-dimensional Ti_3_C_2_T_x_ MXene using HCl + LiF etchant: Enhanced exfoliation and delamination. J. Alloys Compd..

