# Supplementary material for: Rational Design of Flexible, Self-Supporting, and Binder-Free Prussian White/KetjenBlack/MXene Composite Electrode for Sodium-Ion Batteries with Boosted Electrochemical Performance
Source: Molecules. 2024 Jun 27;29(13):3048. doi: 10.3390/molecules29133048 (PMC11243252; doi:10.3390/molecules29133048)
Supplement: Supplementary file 1 [file molecules-29-03048-s001.zip › molecules-3053797-supplementary.pdf]

molecules MDPI

Supplementary Material

Rational Design of Flexible, Self-Supporting, and Binder-Free Prussian White/KetjenBlack/MXene Composite Electrode for Sodium-Ion Batteries with Boosted Electrochemical Performance

Xiaowen Dai [1,†], Jingyun Chun [2,†], Xiaolong Wang [1,*], Tianao Xv [1], Zhengran Wang [3], Chuanliang Wei [4] and Jinkui Feng [3,*]

1 School of Electrical Engineering, Shandong University, Jinan 250061, China
2 Jiaxing Power Supply Company, State Grid Zhejiang Electric Power Co., Ltd., Jiaxing 314000, China
3 Key Laboratory for Liquid-Solid Structural Evolution & Processing of Materials (Ministry of Education), School of Materials Science and Engineering, Shandong University, Jinan 250061, China
4 School of Chemistry and Chemical Engineering, Shandong University, Jinan 250061, China
* Correspondence: wangxiaolong@sdu.edu.cn (X.W.); jinkui@sdu.edu.cn (J.F.)
† These authors contributed equally to this work.

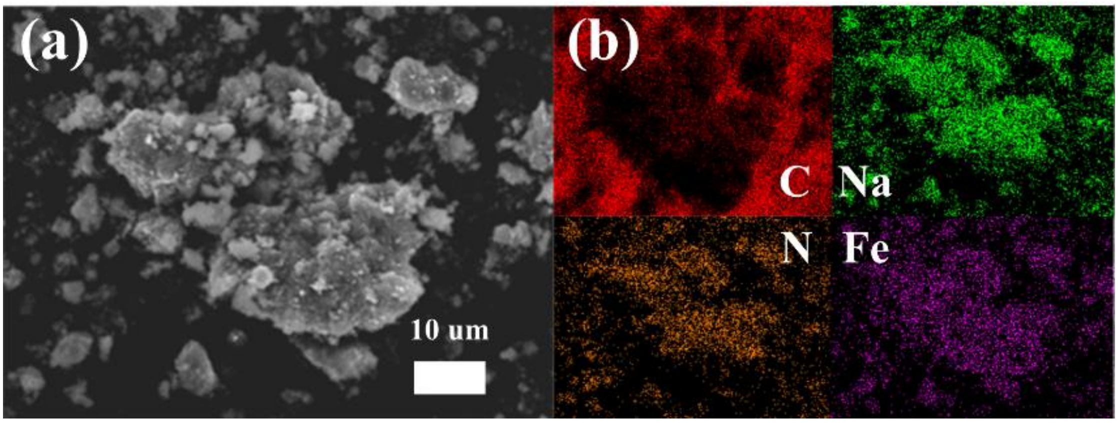

Figure S1 (**a**) SEM image and (**b**) EDS of the PW.

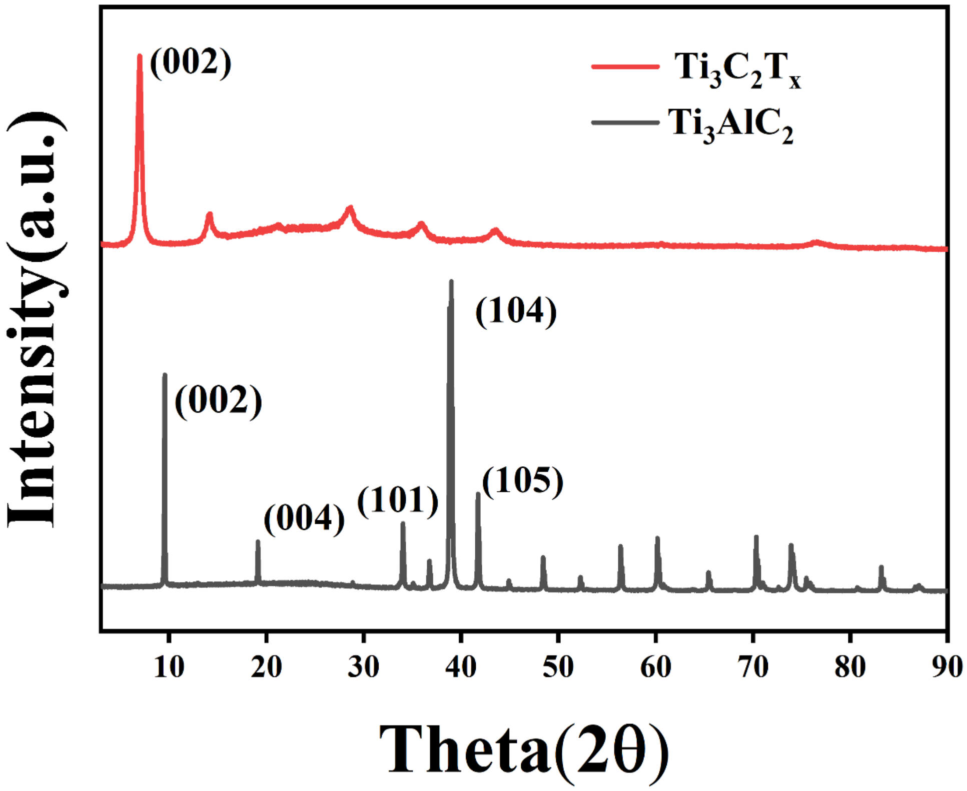

Figure S2 XRD of the Ti_3AlC_2 and $Ti_3C_2T_x$ MXene.

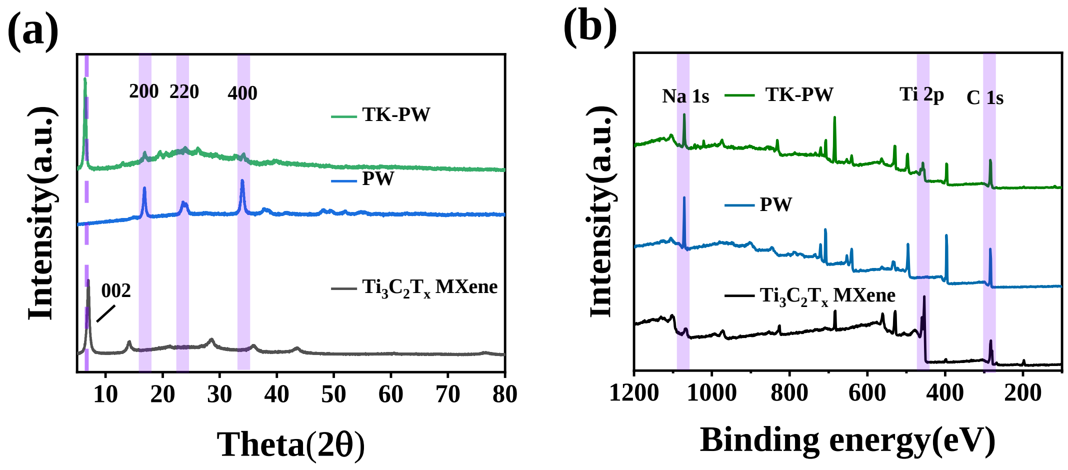

Figure S3 (a) XRD of the $Ti_3C_2T_x$ MXene, PW, and TK–PW. (b) XPS full spectra of the $Ti_3C_2T_x$ MXene, PW, and TK–PW.
